# Supplementary material for: Twinning-assisted dynamic adjustment of grain boundary mobility
Source: Nat Commun. 2021 Nov 18;12:6695. doi: 10.1038/s41467-021-27002-3 (PMC8602286; doi:10.1038/s41467-021-27002-3)
Supplement: Supplementary file 1 — Supplementary Information [file 41467_2021_27002_MOESM1_ESM.pdf]

Twinning-assisted dynamic adjustment of grain boundary mobility

Qishan Huang *et al.*

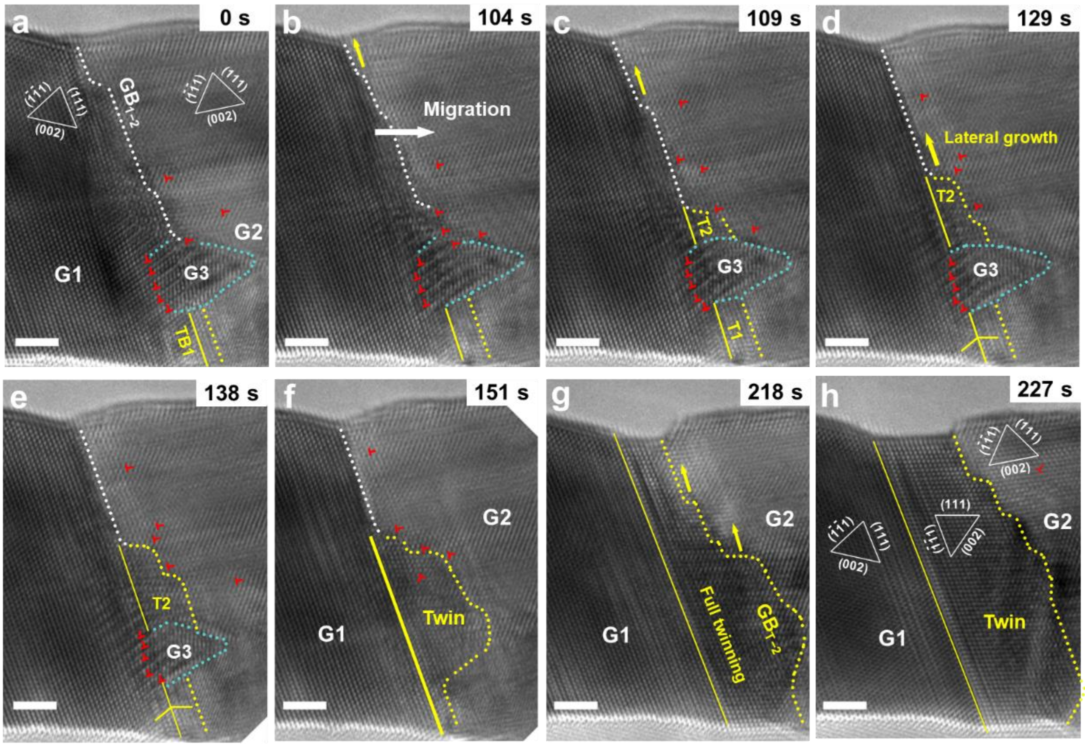

Supplementary Fig. 1 Twinning-assisted migration of GB$_{1\text{-}2}$ with the annihilation of G3. (a) Nucleation of a twin boundary (denoted as TB1) from the intersection of GB$_{1\text{-}2}$ and the bottom free surface, leading to the transformation of the original GB$_{1\text{-}2}$ (white dotted line) to GB$_{T\text{-}2}$ (yellow dotted line). The left GB segment of G3 consisted of several GB dislocations. **(b-c)** Nucleation of a twin (denoted as T2) from the intersection of GB$_{1\text{-}2}$ and G3 in subsequent tensile loading. Lateral motion of pre-existing facets at GB$_{1\text{-}2}$ (indicated by the yellow arrows) contributed to further migration of GB$_{1\text{-}2}$. **(d-e)** Subsequent lateral growth of T2 through the migration of GB$_{T\text{-}2}$, further transforming the residual GB$_{1\text{-}2}$ to GB$_{T\text{-}2}$. Associated with this process was the shrinkage of G3. **(f)** Merging of T1 and T2, resulting in the truncation of G3. **(g-h)** Continuous migration of the newly formed GB$_{T\text{-}2}$ segment via the disconnection mechanism under further tensile loading. The GB$_{T\text{-}2}$ gradually adjusted its shape to a comparatively smooth one **(h)** with the lateral motion of GB facets (i.e. the so-called GB disconnection[1]), as indicated by the yellow arrows. All scale bars: 2 nm.

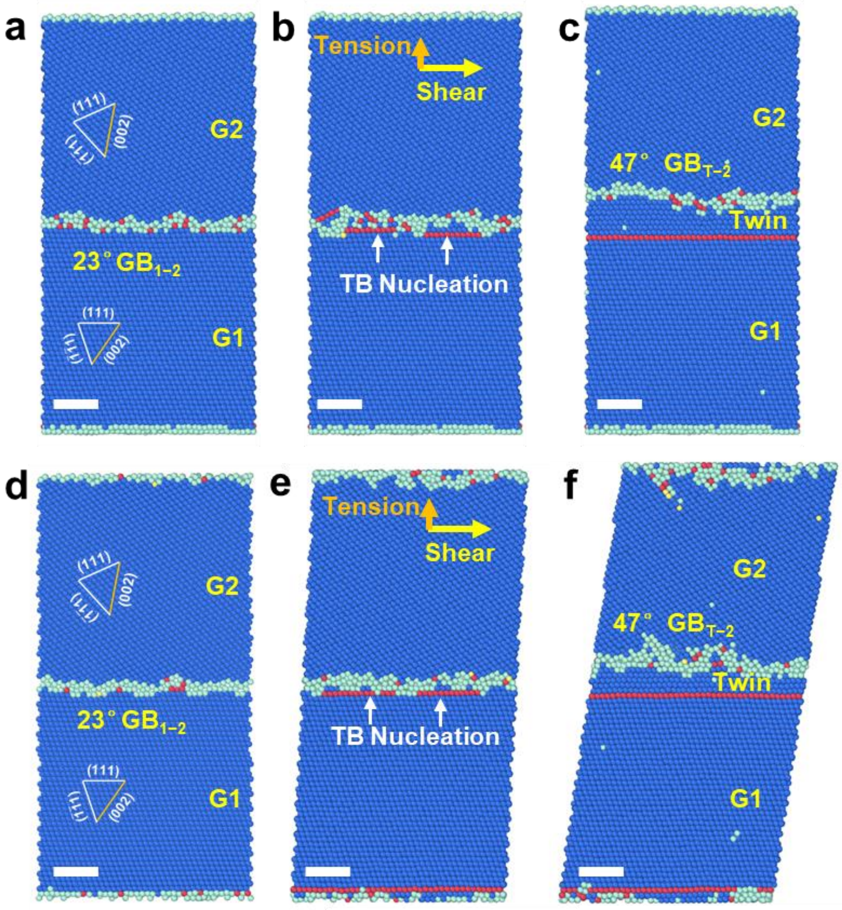

Supplementary Fig. 2 Twinning and GB dynamic adjustment in the simulation model with a horizontal 23° GB subjected to coupled tension and shear loading. Periodic boundary conditions were applied along the GB plane. **(a)** Initial configuration of the 23° GB. **(b)** TB segments nucleation and connection under coupled shear (5×10^7 s^{-1}) and tension (2.5×10^6 s^{-1}). **(c)** The newly formed GB_{T-2} migrated under subsequent combined loading. **(d-f)** Deformation dynamics of the 23° GB under coupled tension and shear in the simulation model with periodic boundary conditions applied in all three dimensions. All scale bars: 2 nm.

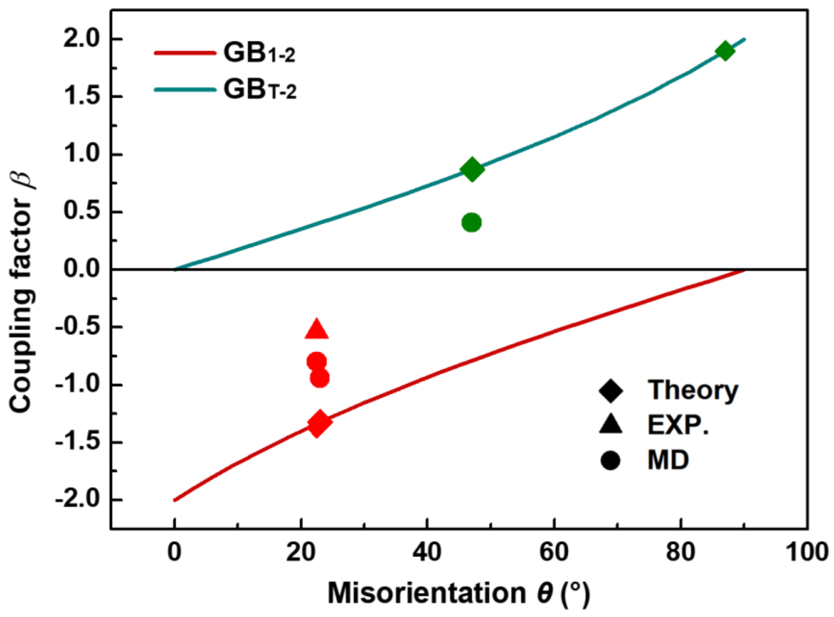

Supplementary Fig. 3 Shear-migration coupling factors of $GB_{1\text{-}2}$ and $GB_{T\text{-}2}$ obtained from theoretical model (see details in Supplementary Discussion 1), experiments and MD simulations.

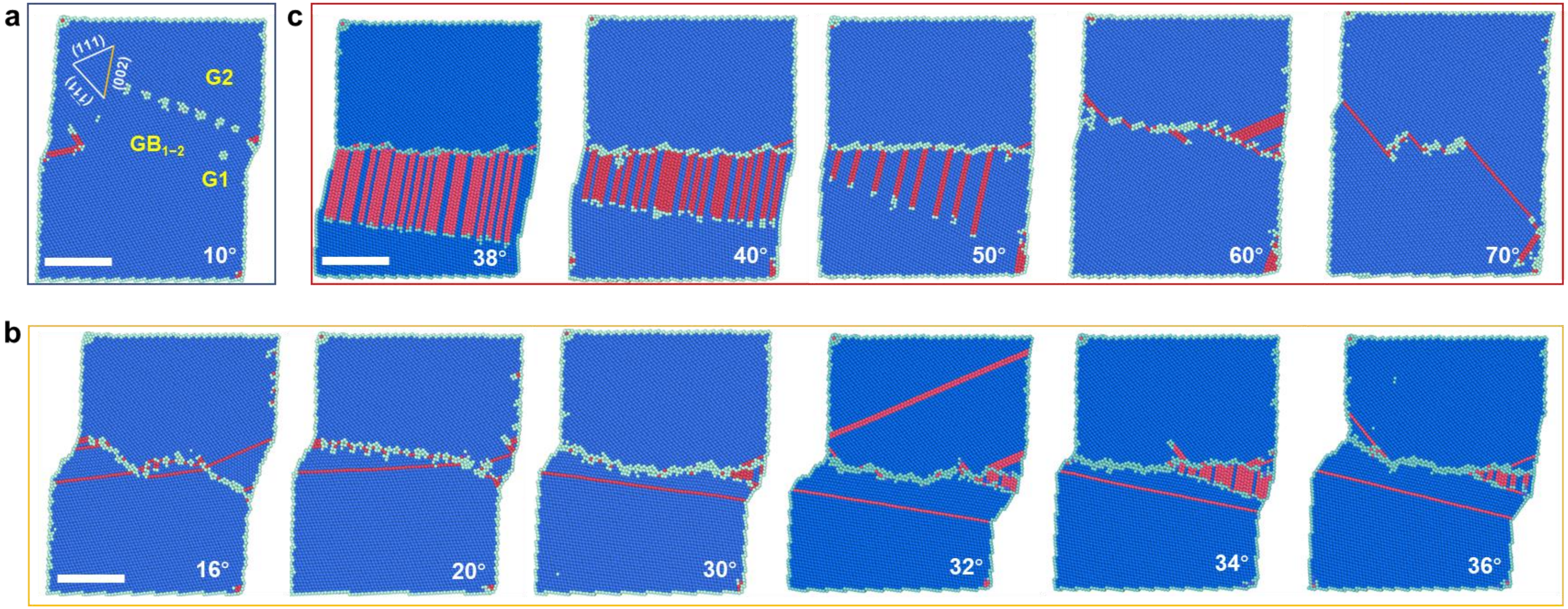

Supplementary Fig. 4 Misorientation effects on the deformation behaviours of GB_{1-2}. The misorientation was varied by rotating the lattice of G1 with respect to the fixed lattice of G2. **(a)** GB deformed by the collective motion of 1/2 <110> dislocations. **(b)** Self-driven dynamic deformation assisted by deformation twinning. Under shearing, the GBs adjusted their structures through decomposition into new GBs and TBs. The newly-formed GBs can migrate smoothly in the subsequent deformation. **(c)** Dislocation slip was activated from the GB. The GB misorientations are marked at the bottom-right of the sample for each case. All scale bars: 5 nm.

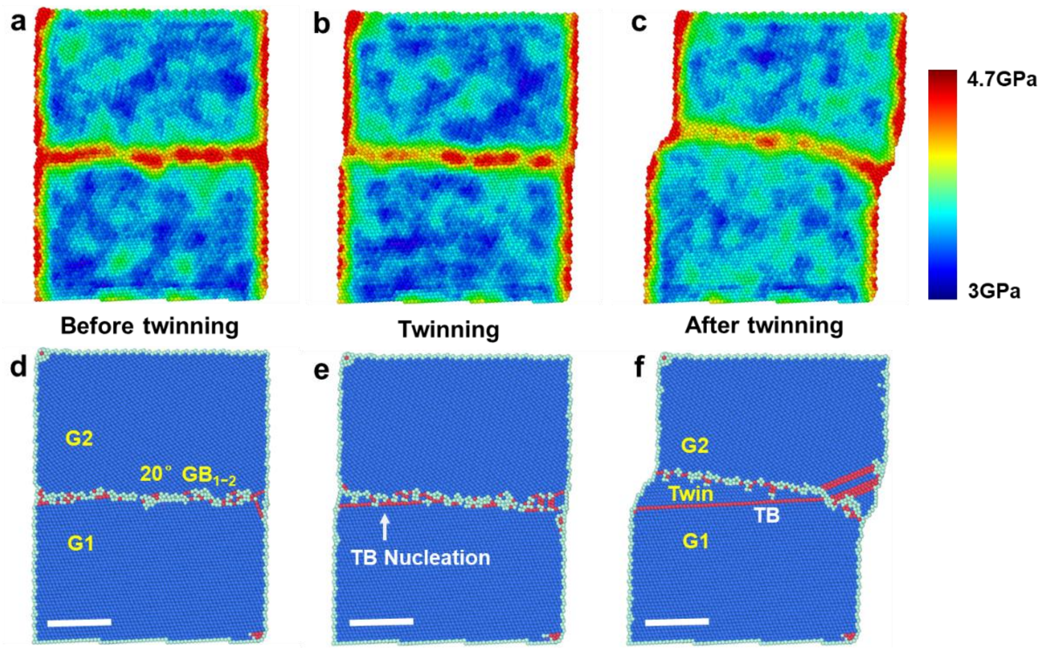

Supplementary Fig. 5 Atomic von Mises stress contours showing evident stress accumulation and release before and after the deformation twinning, respectively. The calculation of the atomic von Mises stress is described in the Methods. The colour is assigned to each atom according to its value of atomic stress. **(a, d)** Before deformation twinning, severe stress accumulation occurred at GB. **(b, e)** Surface-activated twin released the local stress accumulation. **(c, f)** Full twinning induced evident stress reduction at GB. All scale bars: 5 nm.

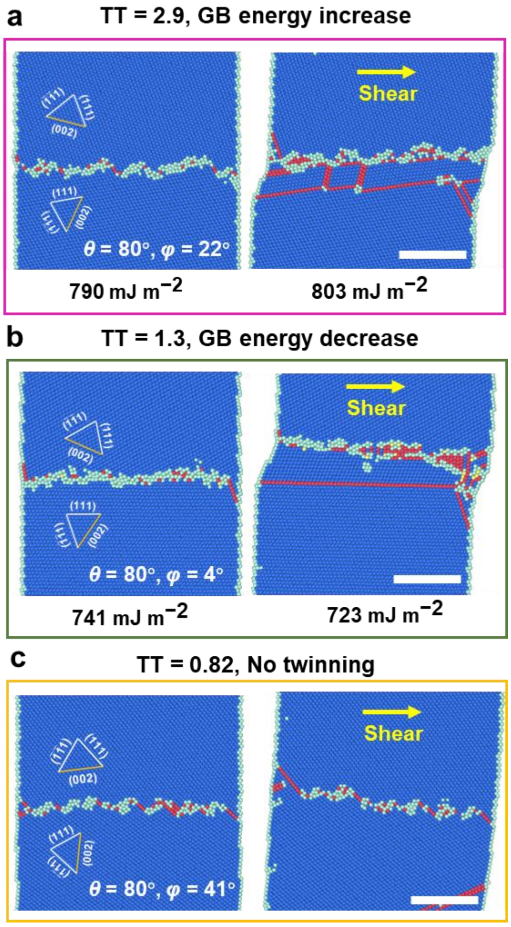

Supplementary Fig. 6 Twinning tendency governed GB deformation. **(a)** GB energy increased after the dynamic structure adjustment for a GB with TT = 2.9. **(b)** GB energy decreased after the dynamic structure adjustment for another GB with TT = 1.3. **(c)** Twinning was absent for a GB with insufficient twinning tendency TT = 0.82. The misorientation and inclination angles are marked at the bottom right of the GB, while the calculated GB energies before and after the twinning-assisted GB adjustment are given at the bottom of the sample. All scale bars: 5 nm.

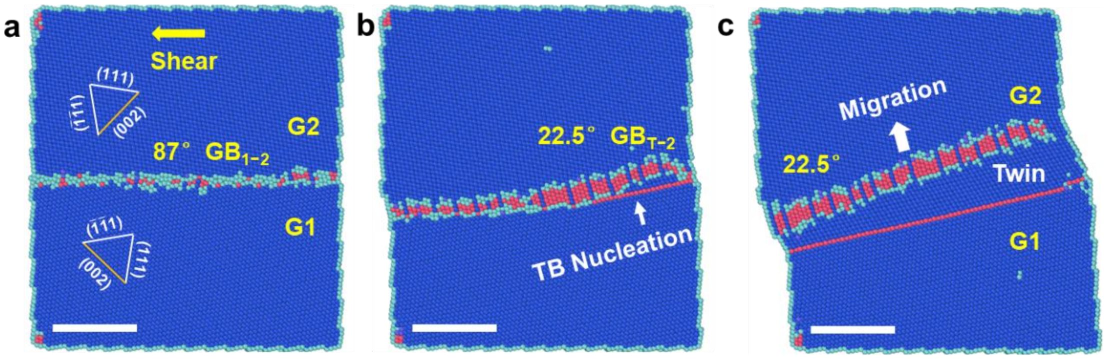

Supplementary Fig. 7 MD simulation of the dynamic adjustment of an 87° <110> tilt GB assisted by twinning. (a) The initial atomic structure of the simulated bicrystal with the same GB_{1-2} misorientation and inclination as that in the experiment (Fig. 4g). **(b)** Self-driven dynamic adjustment of GB via twinning nucleated from right free surface. **(c)** Subsequent smooth migration of the newly formed 22.5° GB_{T-2} by the collective gliding of GB dislocations. All scale bars: 5 nm.

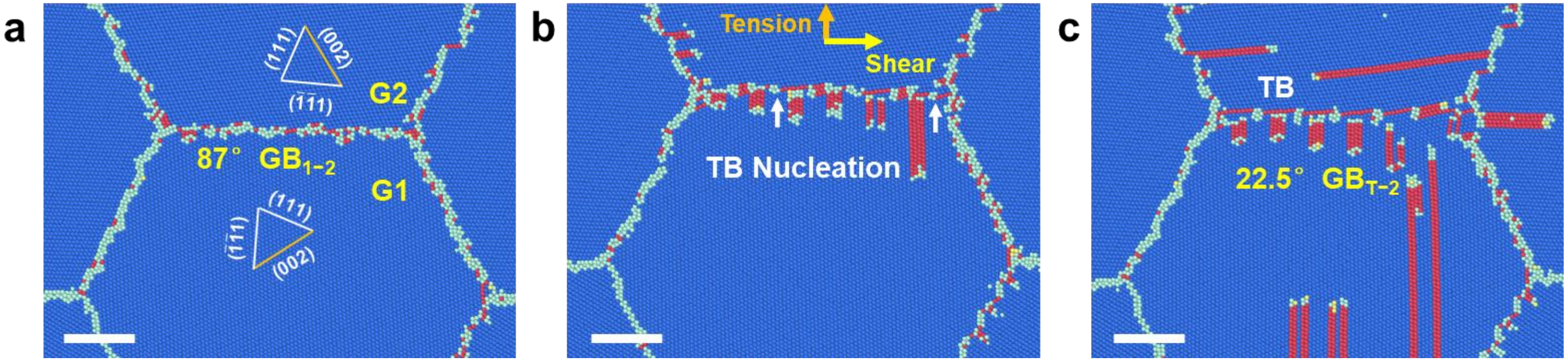

Supplementary Fig. 8 Deformation dynamics of an 87° GB embedded in a polycrystalline Au sample subjected to coupled tension and shear. Periodic boundary conditions were applied in all three directions to avoid free surface effect. **(a)** The initial atomic structure of the simulated polycrystalline Au sample. **(b)** Self-driven dynamic adjustment of GB via deformation twinning. **(c)** Subsequent smooth migration of the newly formed 22.5° $GB_{T\text{-}2}$ by the collective gliding of GB dislocations. All scale bars: 5 nm.

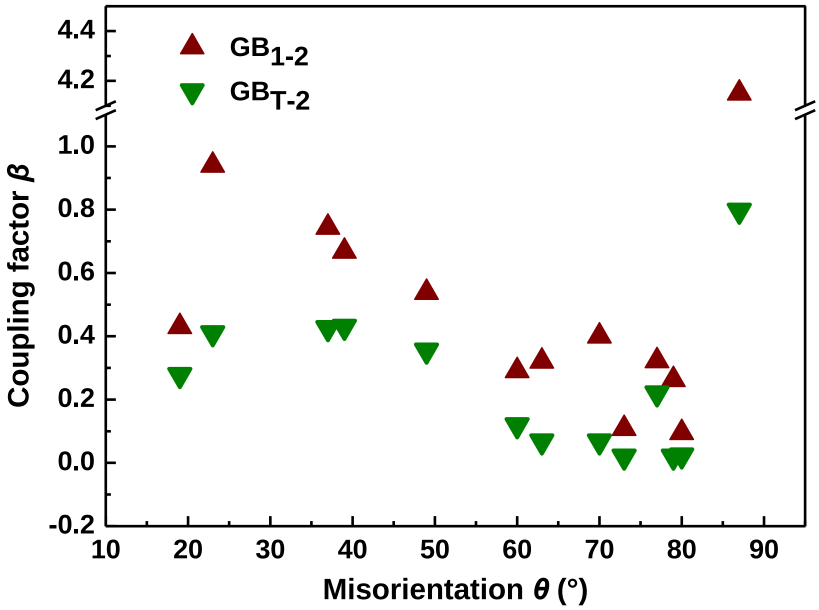

Supplementary Fig. 9 The twinning-assisted variation in shear coupling factor of GBs in nanocrystalline FCC metals (summarized in Fig. 5i). The determination for the misorientation and inclination of the original GB_{1-2} is described in Supplementary Discussion 4. MD simulations were performed on GB_{1-2} and the corresponding GB_{T-2} to calculate the shear coupling factors using the same model as that in Fig. 3a. The shear coupling factor of GB_{T-2} was estimated to be universally lower than GB_{1-2}, indicating an enhanced shear deformability due to deformation twinning.

Supplementary Table 1. The changes of shear coupling factors of GBs with original misorientations of 23° (Figs. 1-2 and Fig. 3a) and 87° (Fig. 4g and Supplementary Fig. 7) due to twinning-assisted dynamic adjustment

Misorientation (°)	Theory	MD	EXP.
23	-1.32	-0.94	/
47	0.87	0.41	/
87	1.9	4.15	/
22.5	-1.32	-0.797	-0.538

Supplementary Discussion 1: Calculation of GB shear-coupling factors

Based on the geometric analysis proposed by Cahn et al.[2], the original GB_{1-2} migrates through the [110] mode, while GB_{T-2} migrates through the [100] mode. The coupling factors ($\beta = v_{//}/v_{\perp}$) for GB_{1-2} and GB_{T-2} can therefore be theoretically predicted by the following formulas:

$$\beta_{<100>} = 2\tan(\frac{\theta}{2}) \tag{1}$$

$$\beta_{<110>} = -2\tan(\frac{\pi}{4} - \frac{\theta}{2}) \tag{2}$$

Following these equations, we plotted the shear coupling factors as a function of the misorientation angle, θ, in Supplementary Fig. 3. Substituting the misorientation angles of GB_{1-2} ($\theta = 23°$) and GB_{T-2} ($\theta = 47°$) into these equations, the magnitude of the theoretical shear coupling factor of GB_{T-2} ($\beta = 0.87$) was found to be smaller than that of GB_{1-2} ($\beta = -1.32$), confirming that GB_{T-2} would migrate a larger distance under the same shearing displacement. We summarized the shear coupling factors obtained from theoretical model, MD simulations and experiments in Supplementary Table 1.

Supplementary Discussion 2: Energy-based elastic driving force for GB migration

As shown in the inset of Fig. 3c, given that a uniaxial tension loading (i.e., a normal strain ε) was applied to the bicrystal to simulate the twinning-assisted dynamic adjustment of GB mobility, which also imposes a biaxial strain state (ε_{xx} and ε_{yy}) in the GB plane (denoted as x-y plane in the following derivation) in order to ensure a steady-state migration without any GB sliding since all resolved shear stresses on the GB plane are zero. We can calculate the corresponding stress components following the Hooke's law:

$$\sigma_{xx} = C_{11}\varepsilon_{xx} + C_{12}\varepsilon_{yy} + C_{13}\varepsilon_{zz} \tag{3}$$

$$\sigma_{yy} = C_{21}\varepsilon_{xx} + C_{22}\varepsilon_{yy} + C_{23}\varepsilon_{zz} \tag{4}$$

$$\sigma_{zz} = C_{31}\varepsilon_{xx} + C_{32}\varepsilon_{yy} + C_{33}\varepsilon_{zz} \tag{5}$$

where the elastic constants can be easily determined using MD simulations. In specific, we applied a finite strain component and measured the variation of the stress tensor, thereby getting the elastic constant matrix. The stored elastic energy density can be written as:

$$F = \frac{1}{2}\sigma_{ij}\varepsilon_{ij} = \frac{1}{2}\sigma_{xx}\varepsilon_{xx} + \frac{1}{2}\sigma_{yy}\varepsilon_{yy} \tag{6}$$

The GB migration velocity can be expressed as $v = MP$, with M and P being the GB mobility and the driving force, respectively. The driving force P can be calculated as $P = F_{+} - F_{-}$, where F_{+} and F_{-} are the elastic energies in the upper and lower grains across the GB, respectively. In our MD simulations, the elastic energy densities of the three single crystalline grains (i.e., G1, G2 and Twin in Fig. 3c) were estimated to be $F_{\mathrm{G1}} = 40.89\varepsilon^2$, $F_{\mathrm{G2}} = 100.75\varepsilon^2$, and $F_{\mathrm{Twin}} = 19.79\varepsilon^2$, respectively. One then arrived

at the driving force $P = 59.86\varepsilon^2$ for $GB_{1\text{-}2}$ and $P = 80.96\varepsilon^2$ for $GB_{T\text{-}2}$.

Supplementary Discussion 3: Critical twinning tendency

As proposed in a previous study by Kim et al[3], the critical stress required to generate partial dislocations can be derived as

$$\tau_c = \frac{\Delta\gamma_{\mathrm{i}}}{b_p} + \frac{Gb_p}{2\pi R}\left(\frac{2-v}{1-v}\right)\ln\left(\frac{8\alpha R}{\mathrm{e}^2 r_0}\right) \tag{7}$$

where $\Delta\gamma_{\mathrm{i}}$ is the energy barrier for the formation of partials, b_p is the Burgers vector along the slip direction, G is the shear modulus, R is the radius of sample, v is the Poisson's ratio, α is a correction factor (~0.5), and r_0 is the core cut-off radius of the dislocation. The energy barrier for the formation of twinning partials and trailing partials can be derived as $\Delta\gamma_{\mathrm{tw}} = \gamma_{\mathrm{utf}} - \gamma_{\mathrm{sf}}$ and $\Delta\gamma_{\mathrm{tr}} = \gamma_{\mathrm{usf}} - \gamma_{\mathrm{sf}}$. They can be obtained from the generalized stacking fault energy curve[4]. Following this method, the critical twinning tendency was calculated to be 0.93 for Au.

Supplementary Discussion 4: Twinning-induced crystallographic adjustment of GBs among different nanocrystalline metals

Numerous observations of parallel or oblique intersections of GBs and TBs were reported in nanocrystalline metals, with a wide range of misorientations and grain sizes. Unlike in bicrystals, GBs in nanocrystalline metals are usually pinned by the GB/TB junctions, so that their self-adjustments are frequently triggered to accommodate the local plasticity. In nanocrystalline with complex GBs networks, deformation twinning is frequently associated with GBs, and is probably formed by the self-stimulated adjustment of GB structure. As a result, GB crystallography adjustment can be identified based on a simple geometric analysis of the resultant GB structure after the designated deformation twinning. When a LAGB (misorientation was less than 16°) composed of highly mobile dislocations is intersected with a TB, the transformation was likely to start from an immobile HAGB (misorientation exceeded 16°) to a mobile LAGB[5,6]. If two GBs at the GB-TB junction were both HAGBs, the original GB is assumed to have a smaller intersection angle with respect to the TB as compared with the newly formed GB[7,8,9,10,11]. If the GBs are not pinned by the TB, meaning that the original GB was fully transformed[12,13,14], we thereby determined the original GB structure based on the twin geometry. Fig. 5i summarized these *in situ* and *ex situ* experimental results associated with the self-driven twinning induced GB adjustment, in samples obtained by different methods.

Supplementary References

1. Zhu Q*, et al.* In situ atomistic observation of disconnection-mediated grain boundary migration. *Nat. Commun.* **10**, 156 (2019).

2. Cahn JW, Mishin Y, Suzuki A. Duality of dislocation content of grain boundaries. *Philos. Mag.* **86**, 3965-3980 (2006).

3. Kim S-H, Park J-H, Kim H-K, Ahn J-P, Whang D-M, Lee J-C. Twin boundary sliding in single crystalline Cu and Al nanowires. *Acta Mater.* **196**, 69-77 (2020).

4. Wang C, Wang H, Huang T, Xue X, Qiu F, Jiang Q. Generalized-stacking-fault energy and twin-boundary energy of hexagonal close-packed Au: A first-principles calculation. *Science* **5**, 10213 (2015).

5. Wang L*, et al.* Grain rotation mediated by grain boundary dislocations in nanocrystalline platinum. *Nat. Commun.* **5**, 4402 (2014).

6. Amin-Ahmadi B*, et al.* Effect of deposition rate on the microstructure of electron beam evaporated nanocrystalline palladium thin films. *Thin Solid Films* **539**, 145-150 (2013).

7. Chen M, Ma E, Hemker KJ, Sheng H, Wang Y, Cheng X. Deformation Twinning in Nanocrystalline Aluminum. *Science* **300**, 1275-1277 (2003).

8. Wu XL*, et al.* New Deformation Twinning Mechanism Generates Zero Macroscopic Strain in Nanocrystalline Metals. *Philos. Mag. Lett.* **100**, 192-195 (2008).

9. Wu XL, Zhu YT. Inverse Grain-Size Effect on Twinning in Nanocrystalline Ni. *Philos. Mag. Lett.* **101**, 025503 (2008).

10. Lu N, Du K, Lu L, Ye HQ. Transition of dislocation nucleation induced by local stress concentration in nanotwinned copper. *Nat. Commun.* **6**, 7648 (2015).

11. Arroyo Rojas Dasilva Y*, et al.* Atomic-scale structural characterization of grain boundaries in epitaxial Ge/Si microcrystals by HAADF-STEM. *Acta Mater.* **167**, 159-166 (2019).

12. Ni S*, et al.* The effect of dislocation density on the interactions between dislocations and twin boundaries in nanocrystalline materials. *Acta Mater.* **60**, 3181-3189 (2012).

13. Luo XM, Zhu XF, Zhang GP. Nanotwin-assisted grain growth in nanocrystalline gold films under cyclic loading. *Nat. Commun.* **5**, 3021 (2014).

14. Li Q, Wang L, Teng J, Pang X, Zou J. In-situ observation of cooperative grain boundary sliding and migration in the nano-twinned nanocrystalline-Au thin-films. *Scr. Mater.* **180**, 97-102 (2020).
