# Supplementary material for: Twinning-assisted dynamic adjustment of grain boundary mobility
Source: Nat Commun. 2021 Nov 18;12:6695. doi: 10.1038/s41467-021-27002-3 (PMC8602286; doi:10.1038/s41467-021-27002-3)
Supplement: Supplementary file 3 — Description of Additional Supplementary Files [file 41467_2021_27002_MOESM3_ESM.pdf]

Description of Additional Supplementary Files

File Name: Supplementary Movie 1
Description: Twinning-assisted dynamic adjustment of an Au bicrystal containing a 23° [1 -10] tilt GB. The structure and mobility of the GB was adjusted to promote GB migration.

File Name: Supplementary Movie 2
Description: Atomistic simulation of a 23° [1 -10] tilt GB in an Au bicrystal demonstrating the atomic-scale structural adjustment during the twinning-assisted GB dynamic transformation under shear loading.

File Name: Supplementary Movie 3
Description: Twinning-assisted dynamic adjustment of an Au bicrystal containing an 87° [1 -10] tilt GB. The structure and mobility of the GB was adjusted to promote GB migration.

File Name: Supplementary Movie 4
Description: Atomistic simulation of an 87° [1 -10] tilt GB in an Au bicrystal demonstrating the atomic-scale structural adjustment during the twinning-assisted GB dynamic transformation under shear loading.
